# Author Correction: A CCG expansion in *ABCD3* causes oculopharyngodistal myopathy in individuals of European ancestry

**DOI:** 10.1038/s41467-024-53151-2

**Published:** 2024-10-17

**Authors:** Andrea Cortese, Sarah J. Beecroft, Stefano Facchini, Riccardo Curro, Macarena Cabrera-Serrano, Igor Stevanovski, Sanjog R. Chintalaphani, Hasindu Gamaarachchi, Ben Weisburd, Chiara Folland, Gavin Monahan, Carolin K. Scriba, Lein Dofash, Mridul Johari, Bianca R. Grosz, Melina Ellis, Liam G. Fearnley, Rick Tankard, Justin Read, Ashirwad Merve, Natalia Dominik, Elisa Vegezzi, Ricardo P. Schnekenberg, Gorka Fernandez-Eulate, Marion Masingue, Diane Giovannini, Martin B. Delatycki, Elsdon Storey, Mac Gardner, David J. Amor, Garth Nicholson, Steve Vucic, Robert D. Henderson, Thomas Robertson, Jason Dyke, Vicki Fabian, Frank Mastaglia, Mark R. Davis, Marina Kennerson, OPDM study group, Piraye Oflazer, Nazli A. Başak, Hülya Kayserili, Gözde Yeşil, Edoardo Malfatti, James B. Lilleker, Matthew Wicklund, Robert D. S. Pitceathly, Stefen Brady, Bernard Brais, David Pellerin, Stephan Zuchner, Matt C. Danzi, Marina Grandis, Giacomo P. Comi, Stefania P. Corti, Elena Abati, Antonio Toscano, Arianna Manini, Arianna Ghia, Cristina Tassorelli, Ilaria Quartesan, Roberto Simone, Alexander M. Rossor, Mary M. Reilly, Liam Carroll, Volker Straub, Bjarne Udd, Zhiyong Chen, Gisèle Bonne, Ros Quinlivan, Simon Hammans, Arianna Tucci, Melanie Bahlo, Catriona A. McLean, Nigel G. Laing, Tanya Stojkovic, Henry Houlden, Michael G. Hanna, Ira W. Deveson, Paul J. Lockhart, Phillipa J. Lamont, Michael C. Fahey, Enrico Bugiardini, Gianina Ravenscroft

**Affiliations:** 1 Department of Neuromuscular Diseases, UCL Queen Square Institute of Neurology, London, UK; 2 Department of Brain and Behavioral Sciences, University of Pavia, Pavia, Italy; 3 Pawsey Supercomputing Research Centre, Kensington, WA Australia; 4 Harry Perkins Institute of Medical Research, Nedlands, WA Australia; 5 Department of Neurology and Instituto de Biomedicina de Sevilla, Hospital Universitario Virgen del Rocío/Universidad de Sevilla/CSIC, Sevilla, 41013 Spain; 6 Genomics and Inherited Disease Program, Garvan Institute of Medical Research, Sydney, NSW Australia; 7 Centre for Population Genomics, Garvan Institute of Medical Research and Murdoch Children’s Research Institute, Sydney, NSW Australia; 8 School of Computer Science and Engineering, University of New South Wales, Sydney, NSW Australia; 9 Program in Medical and Population Genetics, Broad Institute of MIT and Harvard, Cambridge, MA USA; 10 Centre for Medical Research, University of Western Australia, Nedlands, WA Australia; 11 Northcott Neuroscience Laboratory, ANZAC Research Institute, Sydney, NSW 2139 Australia; 12 Faculty of Medicine and Health, University of Sydney, Sydney, NSW 2006 Australia; 13 Population Health and Immunity Division, The Walter and Eliza Hall Institute of Medical Research, 1 G Royal Parade, Parkville, VIC 3052 Australia; 14 Department of Medical Biology, The University of Melbourne, 1G Royal Parade, Parkville, VIC3052 Australia; 15 Department of Mathematics and Statistics, Curtin University, Perth, WA Australia; 16 Bruce Lefroy Centre, Murdoch Children’s Research Institute, Parkville, VIC Australia; 17 Department of Paediatrics, University of Melbourne, Royal Children’s Hospital, Parkville, VIC Australia; 18 Department of Neuropathology, National Hospital for Neurology and Neurosurgery, London, United Kingdom; 19 IRCCS Mondino Foundation, Pavia, Italy; 20 Centre de Référence des Maladies Neuromusculaires Nord-Est-Ile de France, Hôpital Pitié-Salpêtrière, Institut de Myologie, APHP, Paris, France; 21 CHU Grenoble Alpes, Grenoble Institut Neurosciences, INSERM, U1216, Université Grenoble Alpes, Grenoble, France; 22 Neurology Department, The Alfred Hospital, Melbourne, VIC Australia; 23 The Laboratory for Genomic Medicine, University of Otago, Dunedin, New Zealand; 24 Molecular Medicine Laboratory, Concord Repatriation General Hospital, Sydney, NSW 2139 Australia; 25 Brain and Nerve Research Centre, Concord Repatriation General Hospital, Sydney, NSW 2139 Australia; 26 Department of Neurology, Royal Brisbane & Women’s Hospital, Herston, QLD Australia; 27 UQ Centre for Clinical Research, Herston, QLD Australia; 28 Pathology Queensland, Royal Brisbane and Women’s Hospital, Herston, QLD Australia; 29 School of Biomedical Sciences, The University of Queensland, St. Lucia, QLD Australia; 30 PathWest Neuropathology, Royal Perth Hospital, Perth, WA Australia; 31 School of Medicine and Pharmacology, University of Western Australia, Crawley, WA Australia; 32 Perron Institute for Neurological and Translational Science, Nedlands, WA Australia; 33 Neurogenetics Unit, Diagnostic Genomics, PathWest, Nedlands, WA Australia; 34 Dubowitz Neuromuscular Centre, UCL Great Ormond Street Institute of Child Health & MRC Centre for Neuromuscular Diseases, London, United Kingdom; 35 Wessex Neurological Centre, University Hospital Southampton, Southampton, United Kingdom; 36 William Harvey Research Institute, Faculty of Medicine and Dentistry, Queen Mary University of London, London, United Kingdom; 37 Department of Medical Biology, The University of Melbourne, Parkville, Victoria, Australia; 38 Department of Anatomical Pathology, Alfred Hospital, Melbourne, Victoria, Australia; 39 Neurogenetics Unit, Royal Perth Hospital, Perth, WA Australia; 40 Department of Paediatrics Monash Children’s Hospital, Victoria, Australia; 41 Koc University School of Medicine, Department of Neurology, Istanbul, Turkey; 42 Department of Medical Genetics, Istanbul Medical Faculty, Istanbul, Turkey; 43 Centre de Référence de Pathologie Neuromusculaire Nord-Est-Ile-de-France, Hôpital Henri Mondor, APHP, Créteil, France; 44 Université Paris Est, U955, INSERM, IMRB, F-94010 Créteil, France; 45 Manchester Centre for Clinical Neurosciences, Salford Royal Hospital, Northern Care Alliance NHS Foundation Trust, Manchester, UK; 46 UT Health San Antonio, San Antonio, TX USA; 47 Oxford Muscle Service, John Radcliffe Hospital, Level 3, West Wing, Oxford, OX3 9DU UK; 48 Department of Neurology and Neurosurgery, Montreal Neurological Hospital and Institute, McGill University, Montreal, QC Canada; 49 Dr. John T. Macdonald Foundation, Department of Human Genetics and John P. Hussman Institute for Human Genomics, University of Miami, Miller School of Medicine, Miami, FL USA; 50 IRCCS Policlinico San Martino Hospital, Genoa, Italy; 51 Dipartimento Di Neuroscienze, Riabilitazione, Oftalmologia, Genetica e Scienze Materno-Infantili, Università Di Genova, Genoa, Italy; 52 Dino Ferrari Center, Department of Pathophysiology and Transplantation, University of Milan, 20122 Milan, Italy; 53 Neurology Unit, Fondazione IRCCS Ca’ Granda Ospedale Maggiore Policlinico, Milan, Italy; 54 Neuromuscular and Rare Disease Unit, Fondazione IRCCS Ca’ Granda Ospedale Maggiore Policlinico, Milan, Italy; 55 ERN-NMD Center for Neuromuscular Disorders of Messina, Department of Clinical and Experimental Medicine, University of Messina, Messina, Italy; 56 John Walton Muscular Dystrophy Research Centre, Newcastle University Translational and Clinical Research Institute and Newcastle Hospitals NHS Foundation Trust, Newcastle upon Tyne, UK; 57 Tampere Neuromuscular Center, Tampere, Finland; 58 Folkhalsan Research Center, Helsinki, Finland; 59 Department of Neurology, National Neuroscience Institute, Singapore, 308433 Singapore; 60 Sorbonne Université, INSERM, Institut de Myologie, Centre de Recherche en Myologie, Paris, France

**Keywords:** Medical genetics, Neuromuscular disease

Correction to: *Nature Communications* 10.1038/s41467-024-49950-2, published online 27 July 2024

In this article the OPDM study group member James B Lilleker was incorrectly written as James B Lilliker. The original article has been corrected.

