# Supplementary material for: A CCG expansion in ABCD3 causes oculopharyngodistal myopathy in individuals of European ancestry
Source: Nat Commun. 2024 Jul 27;15:6327. doi: 10.1038/s41467-024-49950-2 (PMC11283466; doi:10.1038/s41467-024-49950-2)
Supplement: Supplementary file 1 — Supplementary Information [file 41467_2024_49950_MOESM1_ESM.pdf]

SUPPLEMENTARY INFORMATION

Andrea Cortese[1,2,*], Sarah J Beecroft[3,4,*], Stefano Facchini[2,1], Riccardo Curro[2,1], Macarena Cabrera-Serrano[3], Igor Stevanovski[5,6], Sanjog Chintalaphani[5,6], Hasindu Gamaarachchi[5,6,7], Ben Weisburd[8], Chiara Folland[3,4], Gavin Monahan[3,4], Carolin K Scriba[3], Lein Dofash[3,4], Mridul Johari[3,4], Bianca R Grosz[9,10], Melina Ellis[9,10], Liam G Fearnley[11,12], Rick Tankard[13], Justin Read[14,15], Ash Merve[16], Natalia Dominik[1], Elisa Vegezzi[17], Ricardo P Schnekenberg[2,1], Gorka Fernandez[18], Marion Masingue[18], Diane Giovannini[19], Martin Delatycki[14,15], Elsdon Storey[20], Mac Gardner[21], David Amor[14,15], Garth Nicholson[9,22], Steve Vucic[10,23], Robert D Henderson[24,25], Thomas Robertson[26,27], Jason Dyke[28,29], Vicki Fabian[28], Frank Mastaglia[30], Mark R Davis[31], Marina Kennerson[9,10,22], OPDM study group[$], Ros Quinlivan[32], Simon Hammans[33], Arianna Tucci[34], Melanie Bahlo[11,12], Catriona A McLean[35,36], Nigel G Laing[3,30], Tanya Stojkovic[18], Henry Houlden[1], Michael G Hanna[1], Ira Deveson[5,6], Paul J Lockhart[14,15], Phillipa J Lamont[37], Michael C Fahey[38], Enrico Bugiardini[1,#], Gianina Ravenscroft[3,4,#]

[*] These authors contributed equally

[#] These authors jointly supervised this work

[$] A list of authors and their affiliations appears at the end of the paper

Affiliations

[1]Department of Neuromuscular Diseases, UCL Queen Square Institute of Neurology, London, United Kingdom

[2]Department of Brain and Behavioral Sciences, University of Pavia, Pavia, Italy

[3]Harry Perkins Institute of Medical Research, Nedlands, WA, Australia

[4]Centre for Medical Research, University of Western Australia, Nedlands, WA, Australia

[5]Genomics Pillar, Garvan Institute of Medical Research, Sydney, NSW, Australia

[6]Centre for Population Genomics, Garvan Institute of Medical Research and Murdoch Children's Research Institute, Sydney, New South Wales, Australia

[7]School of Computer Science and Engineering, University of New South Wales, Sydney, NSW, Australia

[8]Program in Medical and Population Genetics, Broad Institute of MIT and Harvard, Cambridge, MA, USA

[9]Northcott Neuroscience Laboratory, ANZAC Research Institute, Sydney, NSW 2139, Australia

[10]Faculty of Medicine and Health, University of Sydney, Sydney, NSW 2006, Australia

[11]Population Health and Immunity Division, The Walter and Eliza Hall Institute of Medical Research,

1G Royal Parade, Parkville, VIC 3052, Australia
[12]Department of Medical Biology, The University of Melbourne, 1G Royal Parade, Parkville, VIC 3052, Australia
[13]Department of Mathematics and Statistics, Curtin University, Perth, Western Australia, Australia
[14]Bruce Lefroy Centre, Murdoch Children's Research Institute, Parkville, VIC, Australia
[15]Department of Paediatrics, University of Melbourne, Royal Children's Hospital, Parkville, VIC, Australia
[16]Department of Neuropathology, National Hospital for Neurology and Neurosurgery, London, United Kingdom
[17]IRCCS Mondino Foundation, Pavia, Italy
[18]Nord/Est/Ile-de-France Neuromuscular Reference Center, Institute of Myology, Pitié-Salpêtrière Hospital, APHP, Paris, France
[19]CHU Grenoble Alpes, Grenoble Institut Neurosciences, INSERM, U1216, Université Grenoble Alpes, Grenoble, France
[20]Neurology Department, The Alfred Hospital, Melbourne VIC, Australia
[21]The Laboratory for Genomic Medicine, University of Otago, Dunedin, New Zealand
[22]Molecular Medicine Laboratory, Concord Repatriation General Hospital, Sydney, NSW 2139, Australia
[23]Brain and Nerve Research Centre, Concord Repatriation General Hospital, Sydney, NSW 2139, Australia
[24]Department of Neurology, Royal Brisbane & Women's Hospital, Herston, QLD, Australia
[25]UQ Centre for Clinical Research, Herston, QLD, Australia
[26]Pathology Queensland, Royal Brisbane and Women's Hospital, Herston, QLD, Australia
[27]School of Biomedical Sciences, The University of Queensland, St Lucia, QLD, Australia
[28]PathWest Neuropathology, Royal Perth Hospital, Perth, WA, Australia
[29]School of Medicine and Pharmacology, University of Western Australia, Crawley, WA, Australia
[30]Perron Institute for Neurological and Translational Science, Nedlands, WA, Australia
[31]Neurogenetics Unit, Diagnostic Genomics, PathWest, Nedlands, WA, Australia
[32]Dubowitz Neuromuscular Centre, UCL Great Ormond Street Institute of Child Health & MRC Centre for Neuromuscular Diseases, London, United Kingdom
[33]Wessex Neurological Centre, University Hospital Southampton, Southampton, United Kingdom
[34]William Harvey Research Institute, Faculty of Medicine and Dentistry, Queen Mary University of London, London, United Kingdom
[35]Department of Medical Biology, The University of Melbourne, Parkville, Victoria, Australia
[36]Department of Anatomical Pathology, Alfred Hospital, Melbourne, Victoria, Australia

[37]Neurogenetics Unit, Royal Perth Hospital, Perth, WA, Australia
[38]Department of Paediatrics Monash Children's Hospital, Victoria, Australia

Corresponding authors
Andrea Cortese, MD PhD
UCL Institute of Neurology
Queen Square House
Queen Square
WC1N 3BG
Email: andrea.cortese@ucl.ac.uk

A/Prof Gianina Ravenscroft
Harry Perkins Institute of Medical Research
6 Verdun St, Nedlands,
WA, Australia, 6009
email: gina.ravenscroft@uwa.edu.au

Keywords: myopathy, *ABCD3*, repeat expansion

SUPPLEMENTARY NOTE 1

ABCD3 repeat expansions are very rare in srWGS and optical genome mapping control datasets

Analysis of 16,442 gnomAD samples using ExpansionHunter found that the repeat size distribution had a mean of 7.1 x CCG repeats and a median of 7 x CCG repeats. The median is also 7 x CCG for each gnomAD subpopulation (ASJ, AMI, AMR, MID, SAS, EAS, NFE, FIN, AFR). The most expanded genotype among the gnomAD samples was 7/44 (CI: 7-7/43-58). The REViewer read visualisation for this sample contained several low-quality read alignments but supported a genotype of at least 38 repeats in the long allele, implying that ExpansionHunter works reasonably well for expansions at this locus **(Figure S2)**.

Two samples in the CMG and RGP cohorts (n=3,270) had expansions longer than any of those found in gnomAD (ie. > 44xCCG). The first sample was an unaffected father of an affected daughter with holoprosencephaly and hearing loss. ExpansionHunter reported his genotype as 7/81 (CI: 7-7/64-105). The other sample is the proband from OPDM family AUS1-V:3 and had a genotype of 7/80 (CI: 7-7/63-106). We have not been able to ascertain any further clinical details for the gnomAD individual with at least 38 repeats or the unaffected father in the RGP cohort). Thus, we do not know if they have an OPDM phenotype.

Analysis of 35,749 WGS from non-neurological controls enrolled in the Genomics England 100,000 Genome Project showed a median size for *ABCD3* repeat of 7 repeats and mean 7.17. EH estimated ten out of 71,498 alleles (0.013%) to have expansions >50 CCG repeat, corresponding to the average read length of 150bp. In all, the estimated repeat number was lower than the two OPDM cases (estimated repeat sizes: 52, 52, 52, 52, 62, 78, 80, 81, 82 and 93 repeats; **Figure 2B**). Genomics England policy does not allow for contact and examination of reportedly unaffected individuals; therefore, we could not obtain more DNA for precise sizing of the repeat or access medical files for these 10 subjects. We performed a similar analysis in the complete cohort of 14,600 neurological patients in the Genomics England 100,000 Genome Project and identified, along with UK1-II-1 and UK2-III-2 OPDM cases, two additional individuals with an estimated repeat length of 61 and 64 repeats. We reviewed the cases and performed long read sequencing in both. The first case was a 75-year-old man affected by pure hereditary spastic paraparesis (HSP), genetically unconfirmed. His brother is also affected by HSP but does not carry CCG expansion in *ABCD3*. Long read sequencing showed 120 uninterrupted CCG repeats. Upon recent evaluation he had no signs of OPDM. The second individual is a 38-year-old lady affected by distal hereditary motor neuropathy and carrying a homozygous c.250G>C,p.Gly84Arg pathogenic variant in *HSPB1*. Long read sequencing of the

ABCD3 locus showed 88 CCG repeats (data not shown). In both individuals the repeat size was lower compared to the smallest pathogenic repeat identified in OPDM cases.

Finally, *ABCD3* expansion was absent from 724 control alleles and 250 alleles from internal non-OPDM samples which underwent optical genome mapping at UCL Institute of Neurology.

SUPPLEMENTARY FIGURES

Supplementary Figure 1: Linkage analysis. Linkage analysis in families AUS1 and AUS2 indicated a maximum multipoint LOD score of 2.98 corresponding to a 24 MB region of Chr1.

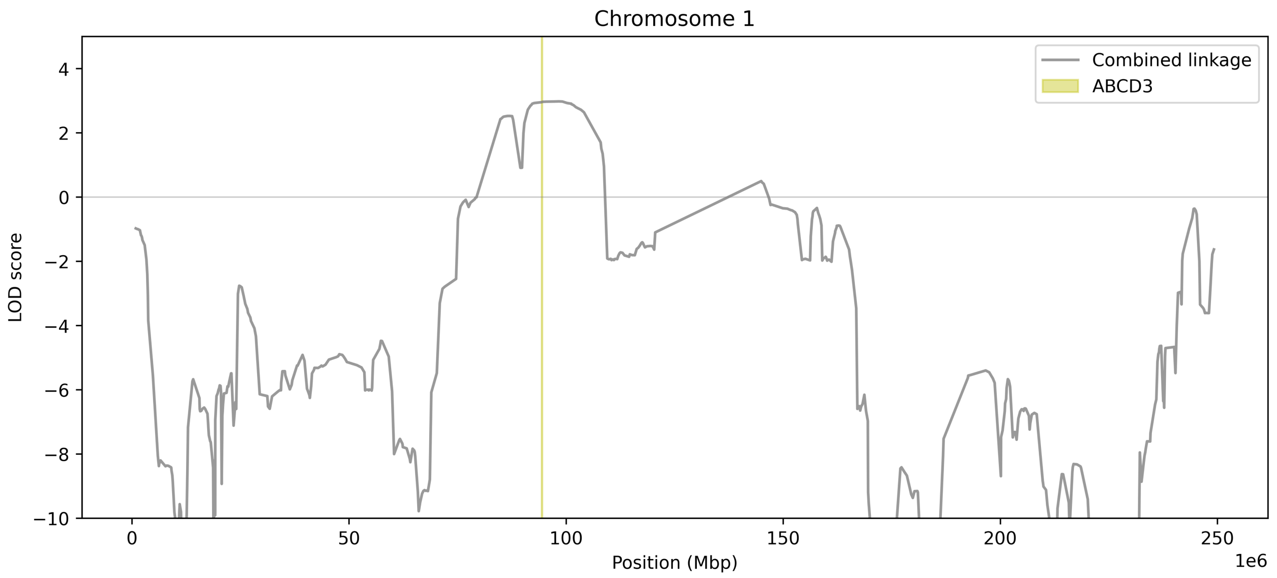

Supplementary Figure 2: Histogram showing the distribution of *ABCD3* repeat sizes detected by ExpansionHunter in the gnomAD cohort.

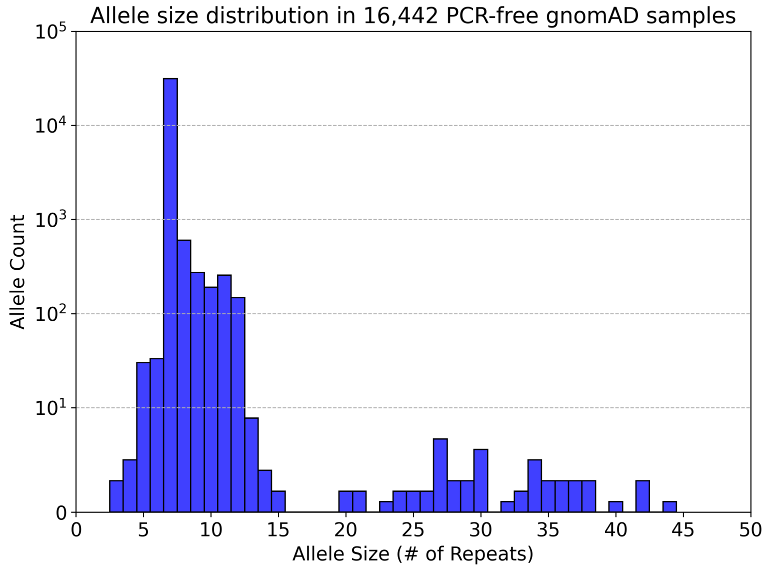

Supplementary Figure 3: Methylation status is unchanged at the *ABCD3* promoter in 17 of 19 affected individuals with CGG expansions, compared to healthy relatives without expanded alleles (marked with an asterisk).

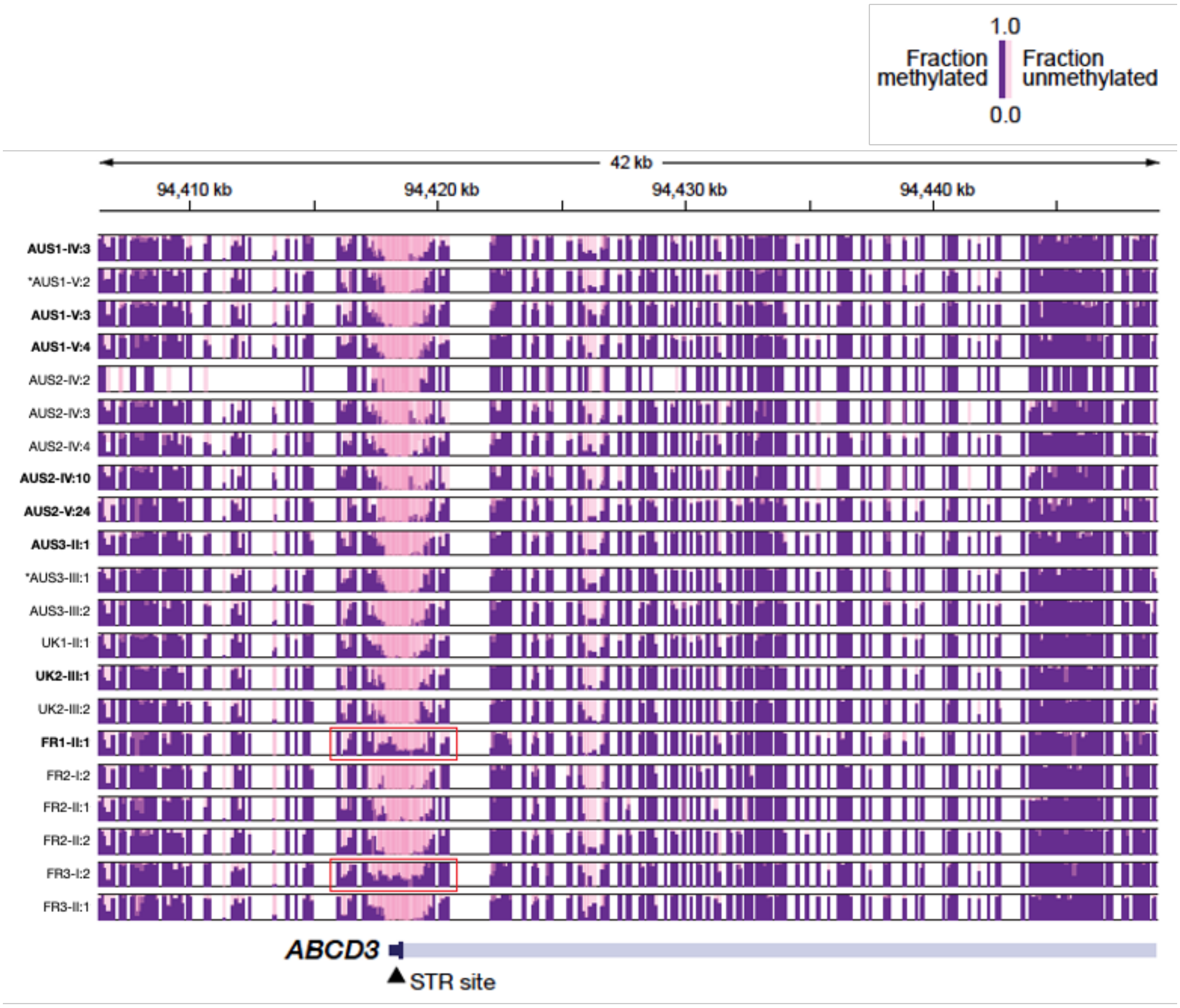

Supplementary Figure 4: Plot of repeat size against age-of-onset in OPDM individuals, showing that larger expansions are associated with an earlier age of onset. The two females shown in purple are the individuals with hypermethylation of their expanded allele, as determined by ONT. There was a weak negative correlation between repeat expansion size and age-of-onset in affected males (y=3.029x+272.8, n=6, p=0.0063) with larger expansions associated with earlier onset of disease. In affected females the age-of-onset is typically ~5 years earlier with no apparent association between repeat expansion size and age-of-onset (y=6.515x+248.3, n=8, p=0.39).

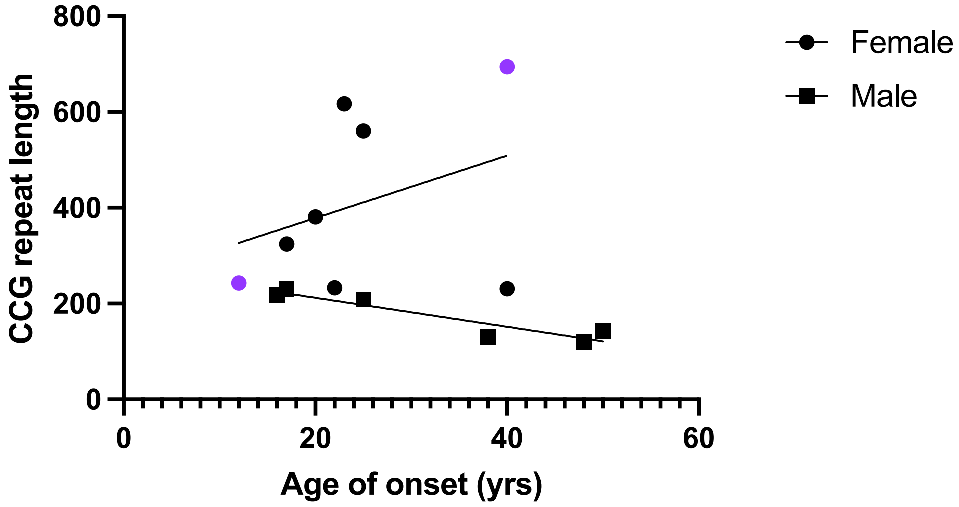

Supplementary Figure 5: Confirmation of the elevated *ABCD3* expression observed in the RNA-seq data by qPCR. qPCR confirmed the findings from RNA-seq that *ABCD3* is expressed at highly levels in OPDM patient muscle compared to healthy controls (p=0.056). The data were generated with primers to the sense transcript of *ABCD3*, there was no amplification using primers designed to the antisense transcript.

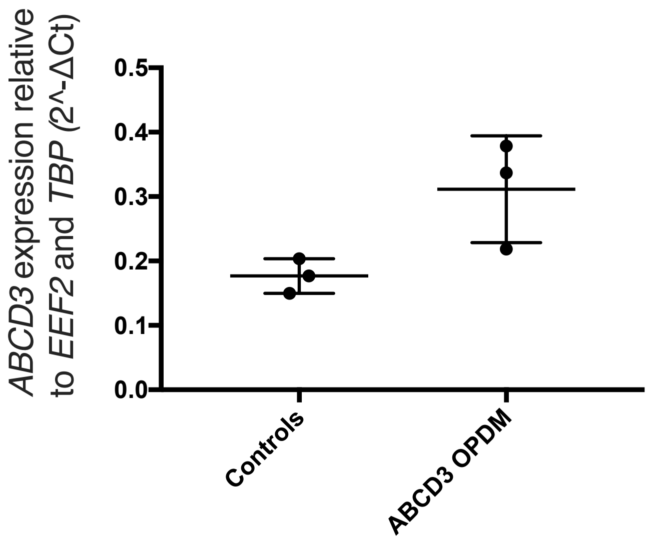

Supplementary Figure 6. FANTOM5 CAGE data for *ABCD3*. In the top pane, the *ABCD3* MANE transcript is highlighted in pink. In the middle pane, CAGE signal for the sense (green) and antisense (purple) direction. In the bottom pane, main CAGE peaks (detected only in the sense direction). Data are taken from the Human hg38 Promoterome in FANTOM5 ZENBU website [https://fantom.gsc.riken.jp/zenbu/].

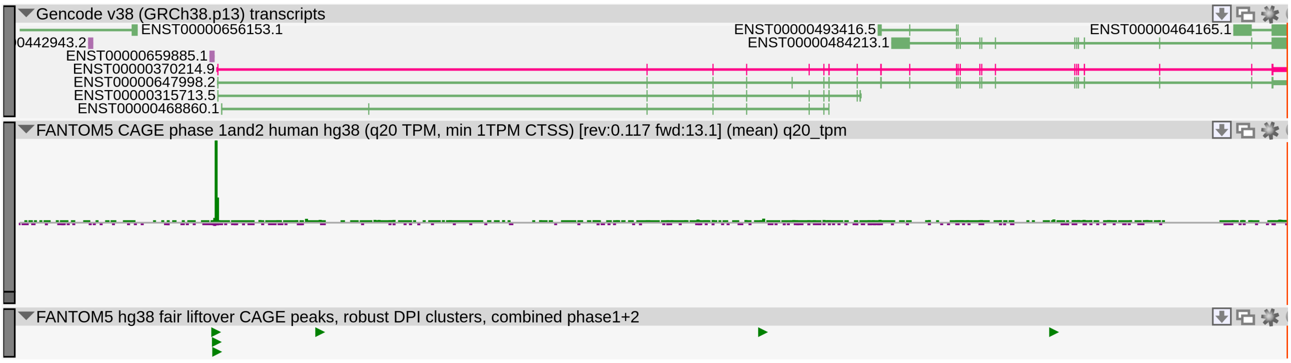

Supplementary Figure 7: A single intranuclear CCG aggregate identified by RNA FISH in a section of fresh-frozen skin from UK2-III:2. Scale bar 10 uM.

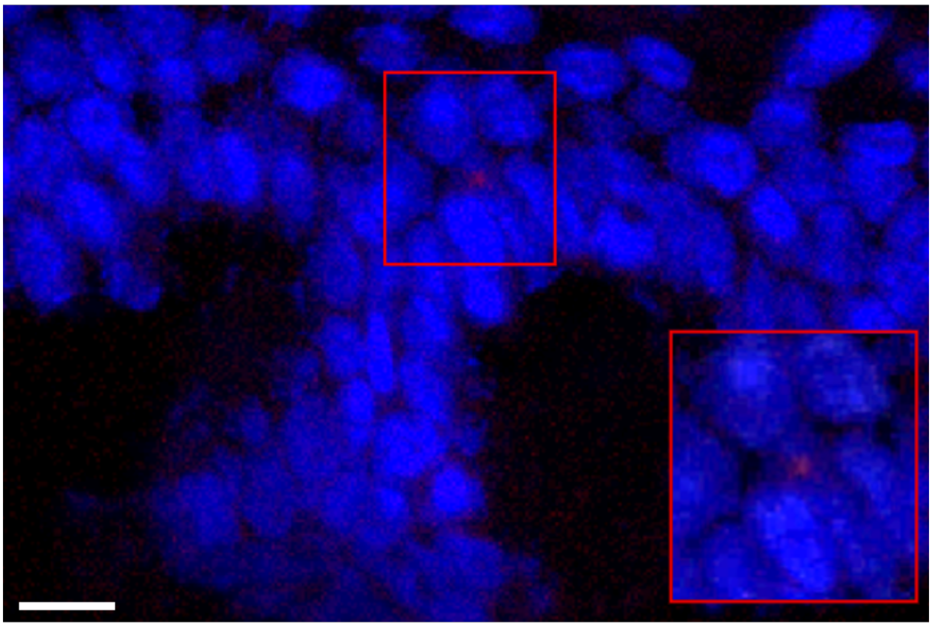

Supplementary Figure 8: Schematic and table showing possible RAN-translation off the *ABCD3* CCG repeat expansion as predicted using the method outlined in Gleason *et al.* 2022 (1)

5’ sequences of *ABCD3* showing (**a**) non-expanded reference sequence mRNA with (**b**) sense strand and (**c**) anti-sense strand mRNA containing triplet repeat expansions. The translation initiation sites (TIS) are indicated for poly-alanine (light blue) and poly-glycine (green). Supplementary Figure 8 was created with BioRender.com released under a Creative Commons Attribution-NonCommercial-NoDerivs 4.0 International license

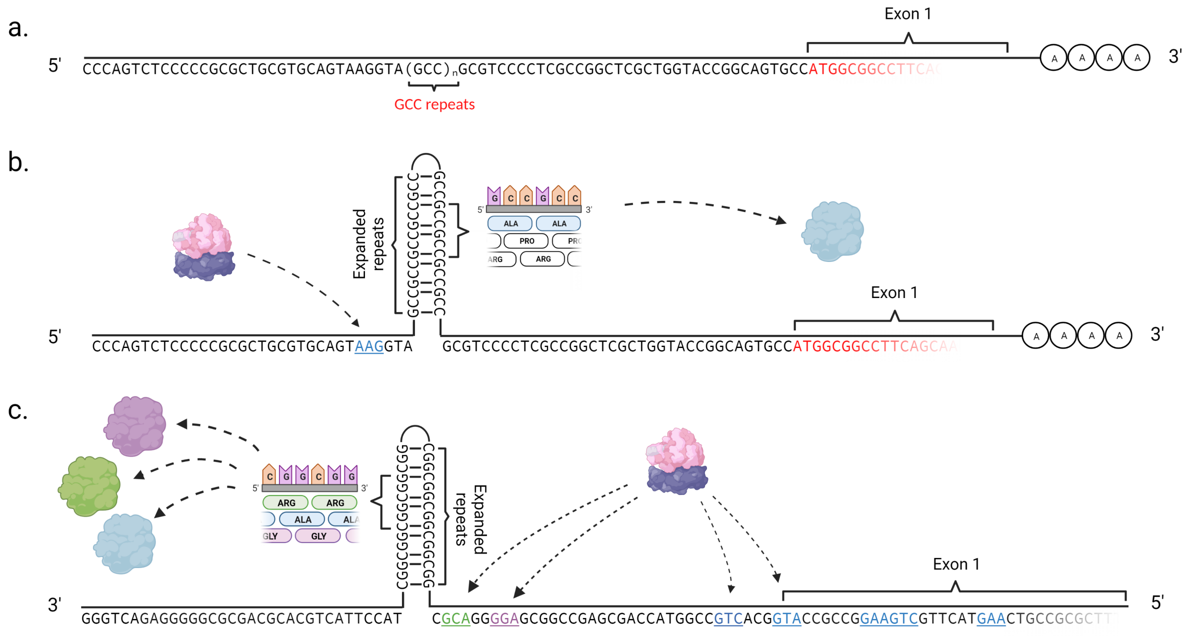

Strand	Trinucleotide repeat	AA	TIS	Distance from repeat	Kozak Similarity Score
Sense	CCG	Pro	N/A	N/A	N/A
	CGC	Arg	N/A	N/A	N/A
	GCC	Ala	AAG	-6	0.74
Antisense	CGG	Arg	N/A	-7	0.65
	GCG	Ala	CTG	-35	0.66
			ATG	-41	0.86
			AAG	-50	0.62
			CTG	-53	0.53
			AAG	-62	0.71
			AGG	-101	0.77
			AGG	-137	0.64
	GGC	Gly	AGG	-12	0.74
			GTG	-120	0.56

SUPPLEMENTARY TABLES

Supplementary Table 1. Clinical features of patients with CGG • CCG expansion causing OPDM1-5

	**OPDM1 (n=4)**	**OPDM2 (n=27)**	**OPDM3 (n=8)**	**OPDM4 (n=11)**	**OPDM5 (n=24)**
Repeat containing gene	*LRP12*	*GIPC1*	*NOTCH2NLC*	*RILPL1*	*ABCD3*
Repeat motif (sense transcript)	*CGG*	*CGG*	*CGG*	*CCG*	*CCG*
Sex (male/female)	3/1	18/9	3/5	7/4	12/12
Age of onset (y ± SD)	32.7 ± 4.6	29.1 ± 10.3	23.1 ± 6.0	23.8 ± 6.2	24.2 ± 11.6
Ptosis	3/4 (75%)	21/25 (84%)	8/8 (100%)	10/10 (100%)	23/23 (100%)
External ophthalmoplegia	3/4 (75%)	17/25 (68%)	5/8 (62.5%)	8/10 (80%)	14/19 (73.6%)
Facial muscle weakness	3/4 (75%)	24/25 (96%)	8/8 (100%)	9/10 (90%)	16/20 (80%)
Dysphagia	3/4 (75%)	18/25 (72%)	7/8 (87.5%)	8/10 (80%)	18/22 (81.8%)
Distal limb weakness	4/4 (100%)	25/25 (100%)	8/8 (100%)	10/10 (100%)	17/22 (77.2%)

SUPPLEMENTARY REFERENCES

1. Gleason AC, Ghadge G, Chen J, Sonobe Y, Roos RP. Machine learning predicts translation initiation sites in neurologic diseases with nucleotide repeat expansions. PLoS One. 2022;17(6):e0256411.
