# Supplementary material for: A CCG expansion in ABCD3 causes oculopharyngodistal myopathy in individuals of European ancestry
Source: Nat Commun. 2024 Jul 27;15:6327. doi: 10.1038/s41467-024-49950-2 (PMC11283466; doi:10.1038/s41467-024-49950-2)
Supplement: Supplementary file 3 — Description of Additional Supplementary Files [file 41467_2024_49950_MOESM3_ESM.pdf]

Description of Additional Supplementary Files

File Name: Supplementary Movie 1

Description: Key finding of neurological examination of subject UK1-II:1
