# Supplementary material for: A CCG expansion in ABCD3 causes oculopharyngodistal myopathy in individuals of European ancestry
Source: Nat Commun. 2024 Jul 27;15:6327. doi: 10.1038/s41467-024-49950-2 (PMC11283466; doi:10.1038/s41467-024-49950-2)
Supplement: Supplementary file 5 — Reporting Summary [file 41467_2024_49950_MOESM5_ESM.pdf]

nature portfolio

Corresponding author(s): Andrea Cortese, Gina Ravenscroft

Last updated by author(s): Feb 15, 2024

Reporting Summary

Nature Portfolio wishes to improve the reproducibility of the work that we publish. This form provides structure for consistency and transparency in reporting. For further information on Nature Portfolio policies, see our Editorial Policies and the Editorial Policy Checklist.

Statistics

For all statistical analyses, confirm that the following items are present in the figure legend, table legend, main text, or Methods section.

n/a	Confirmed	
☐	☒	The exact sample size (*n*) for each experimental group/condition, given as a discrete number and unit of measurement
☐	☒	A statement on whether measurements were taken from distinct samples or whether the same sample was measured repeatedly
☐	☒	The statistical test(s) used AND whether they are one- or two-sided<br>*Only common tests should be described solely by name; describe more complex techniques in the Methods section.*
☐	☒	A description of all covariates tested
☐	☒	A description of any assumptions or corrections, such as tests of normality and adjustment for multiple comparisons
☐	☒	A full description of the statistical parameters including central tendency (e.g. means) or other basic estimates (e.g. regression coefficient) AND variation (e.g. standard deviation) or associated estimates of uncertainty (e.g. confidence intervals)
☐	☒	For null hypothesis testing, the test statistic (e.g. *F*, *t*, *r*) with confidence intervals, effect sizes, degrees of freedom and *P* value noted<br>*Give P values as exact values whenever suitable.*
☒	☐	For Bayesian analysis, information on the choice of priors and Markov chain Monte Carlo settings
☒	☐	For hierarchical and complex designs, identification of the appropriate level for tests and full reporting of outcomes
☒	☐	Estimates of effect sizes (e.g. Cohen's *d*, Pearson's *r*), indicating how they were calculated

Our web collection on statistics for biologists contains articles on many of the points above.

Software and code

Policy information about availability of computer code

Data collection	ABI-3730XL Sequencer, Novaseq 6000, Oxford Nanopore Technologies (ONT) MinION, Saphyr® System (Bionano), QuantStudio™ 6 Pro Real-Time PCR System, inverted fluorescent microscope (model IX-71, Olympus) with a digital camera (model DP-74, Olympus), Zeiss LSM 710 confocal microscope.
Data analysis	SHAPEITv4, Expansion Hunter v3, Expansion Hunter de novo, Bionano Solve 3.6, Bionano Access 3.6, MERLIN, nf-core/rnaseq v3.8.1, Geneious Prime (Biomatters Ltd), ReadFish, slow5tools (v0.3.0), minimap2 (v2.14-r883), Tandem Repeats Finder, F5C (v1.1), STAR (version 2.7.10a), DROP (version 1.3.3), OUTRIDER.

For manuscripts utilizing custom algorithms or software that are central to the research but not yet described in published literature, software must be made available to editors and reviewers. We strongly encourage code deposition in a community repository (e.g. GitHub). See the Nature Portfolio guidelines for submitting code & software for further information.

Data

Policy information about availability of data

All manuscripts must include a data availability statement. This statement should provide the following information, where applicable:

- Accession codes, unique identifiers, or web links for publicly available datasets
- A description of any restrictions on data availability
- For clinical datasets or third party data, please ensure that the statement adheres to our policy

The sequencing data obtained are not publicly available because genetic data are protected by the Personal Information Protection Law, availability of these data is under the regulation by the institutional review board. The data obtained from RNA-seq have been deposited on the European Genome-Phenome Archive (EGA) repository under accession no. EGAS50000000298

Research involving human participants, their data, or biological material

Policy information about studies with human participants or human data. See also policy information about sex, gender (identity/presentation), and sexual orientation and race, ethnicity and racism.

Reporting on sex and gender	Sex has been considered in the study design, study finding apply to both sexes. Among ABCD3 positive patients there were 11 affected males and 13 affected females
Reporting on race, ethnicity, or other socially relevant groupings	Ethnicity was considered in the study design. Genetic data from patients from multiple multiple ethnicities were analyzed, however ABCD3 expansion were only found in Europeans (Caucasians). Ethnicity was both self reported and, for individuals with WGS available, based on genetic data clustering.
Population characteristics	Age, Age of onset, sex, diagnosis, ABCD3 repeat expansion size
Recruitment	Subjects were recruited among patients affected by genetically unconfirmed myopathy by the participating centres in Australia and France or through the Genomics England 100,000 Genome Project.There was no selection bias
Ethics oversight	Human Research Ethics Committee of the University of Western Australia (RA/4/20/1008), the Human Research Ethics Committee of the Royal Children's Hospital (HREC 28097) and Northeast-Newcastle & North Tyneside 1 Research Ethics Committee (22/NE/0080)

Note that full information on the approval of the study protocol must also be provided in the manuscript.

Field-specific reporting

Please select the one below that is the best fit for your research. If you are not sure, read the appropriate sections before making your selection.

☒ Life sciences ☐ Behavioural & social sciences ☐ Ecological, evolutionary & environmental sciences

For a reference copy of the document with all sections, see nature.com/documents/nr-reporting-summary-flat.pdf

Life sciences study design

All studies must disclose on these points even when the disclosure is negative.

Sample size	All available samples diagnosed clinically with OPDM or genetically unconfirmed myopathy were recruited from participating centres in Australia, the United Kingdom (UK), France and through a network of collaborators worldwide (OPDM study group).
Data exclusions	No data were excluded from the analysis
Replication	All experiments excluding high-throughput sequencing or optical genome mapping were repeated independently twice and all attempts at replication were successful.
Randomization	Randomization is not relevant to this study. Both patients with myopathy and controls were tested for the presence of CCG expansions in ABCD3 gene
Blinding	Examiners were blind when performing the quantification of RNAfoci. For the other experiements blinding was not possible because the disease is clinically manifest when examining patients. Also biopsies are processed for clinical purposes and anonymyzation is not possible.

Reporting for specific materials, systems and methods

We require information from authors about some types of materials, experimental systems and methods used in many studies. Here, indicate whether each material, system or method listed is relevant to your study. If you are not sure if a list item applies to your research, read the appropriate section before selecting a response.

Materials & experimental systems

n/a	Involved in the study
☐	☒ Antibodies
☒	☐ Eukaryotic cell lines
☒	☐ Palaeontology and archaeology
☒	☐ Animals and other organisms
☐	☒ Clinical data
☒	☐ Dual use research of concern
☒	☐ Plants

Methods

n/a	Involved in the study
☒	☐ ChIP-seq
☒	☐ Flow cytometry
☒	☐ MRI-based neuroimaging

Antibodies

Antibodies used	Mouse monoclonal antibody against p62 (1:50 dilution, Supplier: Abcam, Cat no. ab56414, Clone 2C11, Lot no. 1013082-1<br><br>Goat anti-mouse IgG2a AlexaFluor™ 555 secondary antibody (1:500 dilution, Supplier: ThermoFisher, Cat no. A21137, Lot no. 2551337
Validation	As is stated on the manufacturers' website, each primary antibody has been individually validated to react against human protein. Validation for all antibodies used for immunohistochemistry has been performed in ISO15189 accredited laboratory.

Clinical data

Policy information about clinical studies

All manuscripts should comply with the ICMJE guidelines for publication of clinical research and a completed CONSORT checklist must be included with all submissions.

Clinical trial registration	N/A
Study protocol	N/A
Data collection	Clinical data of affected individuals were collected from participating centres between January 2021 and September 2023
Outcomes	No predefined outcome was used. Age of disease onset and sex were correlated with repeat length

Plants

Seed stocks	NA
Novel plant genotypes	NA
Authentication	NA
